# Transplant Tolerance, Not Only Clonal Deletion

**DOI:** 10.3389/fimmu.2022.810798

**Published:** 2022-04-21

**Authors:** Bruce M. Hall, Nirupama D. Verma, Giang T. Tran, Suzanne J. Hodgkinson

**Affiliations:** Immune Tolerance Laboratory, School of Medicine, University of New South Wales (UNSW) Sydney, Ingham Institute, and Renal Service and Multiple Sclerosis Clinic, Liverpool Hospital, Liverpool, NSW, Australia

**Keywords:** clonal deletion, graft versus host disease, transplant tolerance, regulatory T (Treg) cells, chimerism

## Abstract

The quest to understand how allogeneic transplanted tissue is not rejected and how tolerance is induced led to fundamental concepts in immunology. First, we review the research that led to the Clonal Deletion theory in the late 1950s that has since dominated the field of immunology and transplantation. At that time many basic mechanisms of immune response were unknown, including the role of lymphocytes and T cells in rejection. These original observations are reassessed by considering T regulatory cells that are produced by thymus of neonates to prevent autoimmunity. Second, we review “operational tolerance” induced in adult rodents and larger animals such as pigs. This can occur spontaneously especially with liver allografts, but also can develop after short courses of a variety of rejection inhibiting therapies. Over time these animals develop alloantigen specific tolerance to the graft but retain the capacity to reject third-party grafts. These animals have a “split tolerance” as peripheral lymphocytes from these animals respond to donor alloantigen in graft versus host assays and in mixed lymphocyte cultures, indicating there is no clonal deletion. Investigation of this phenomenon excludes many mechanisms, including anti-donor antibody blocking rejection as well as anti-idiotypic responses mediated by antibody or T cells. This split tolerance is transferred to a second immune-depleted host by T cells that retain the capacity to effect rejection of third-party grafts by the same host. Third, we review research on alloantigen specific inhibitory T cells that led to the first identification of the CD4^+^CD25^+^T regulatory cell. The key role of T cell derived cytokines, other than IL-2, in promoting survival and expansion of antigen specific T regulatory cells that mediate transplant tolerance is reviewed. The precise methods for inducing and diagnosing operational tolerance remain to be defined, but antigen specific T regulatory cells are key mediators.

## The Origin of the Clonal Deletion Theory of Transplant Tolerance

For over 60 years, the concept of clonal deletion has dominated the field of immunology and the quest for acceptance of transplanted tissue without ongoing immunosuppression. The clonal theory for immune cells and the concept that during ontogeny self-reactive clones are deleted, was made at a time when the function of lymphocytes and the existence of T cells was not appreciated. Because of this, most clinical attempts to induce transplant tolerance aim to delete specific alloreactive cells and the establishment of lympho-haemopoetic chimerism.

Transplant tolerance can be induced in the presence of clones reactive to the graft and in the absence of lympho-haemopoietic chimerism, however. There are many animal models of operational tolerance, where grafts continue to function without immunosuppressive therapy. *Ex vivo* expanded Treg promote tolerance induction (1). In most there is no deletion of alloreactive clones.

This review revisits the findings that led to the theory of clonal deletion and transplant tolerance and describes innumerable mechanisms that control the rejection of allografts without deletion of alloreactive clones. A variety of models of operational tolerance are described, including the spontaneous acceptance of liver grafts and the induction of specific unresponsiveness in murine and swine models by short-term therapy to minimize early rejection. These models do not produce clonal deletion. This review focuses on the induction of alloantigen specific T regulatory cells (Treg) and their role in the generation of “Operational Tolerance” to allografts. These forms of operational tolerance raise the possibility that attempts at clonal deletion have confused the field and may be misguided.

### Self and Non Self

**Figure 1:**
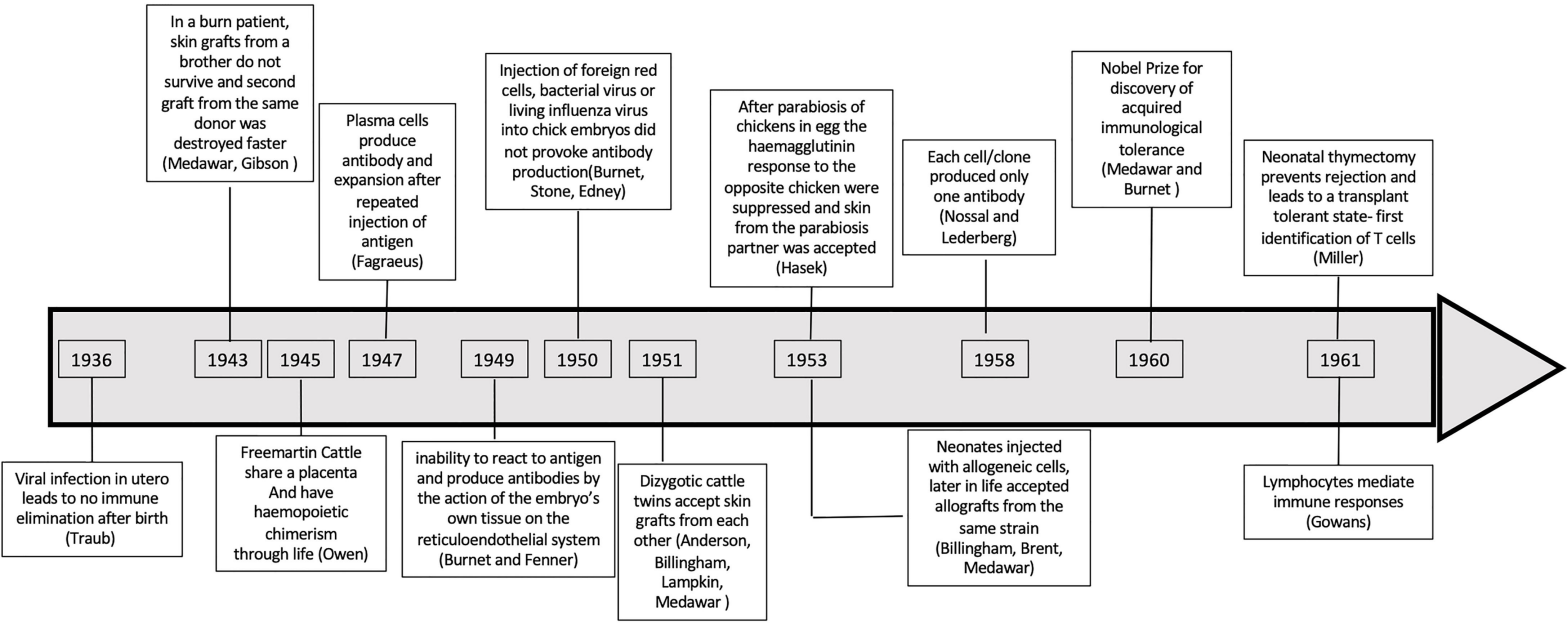
Timeline of observations that supported clonal deletion *in utero* and in neonates. A chronological representation of studies that led to establishment of clonal deletion to explain tolerance.

In 1949, Burnet and Fenner sought to explain why antibody was not generated against self-antigens (2) and how foreign antigen was recognized as non self. They proposed that “self” was defined during embryonic development. The key observations that led Burnet to propose the clonal deletion hypothesis are summarized in **Figure 1** and **Supplementary Table 1**. First was Owen’s observations in dizygotic bovine twin calves who share a placenta *in utero* causing cross circulation of blood. These twins throughout life share each other’s red cell groups (3, 4). These twins produce red cells of their twin, as well as their own red cells, and are haemopoietic chimeras. The Clonal Deletion theory was also supported by an earlier observation by Taube who reported that viral infections acquired *in utero* did not induce antibodies to the virus, whereas mice infected postpartum eliminated the virus (2, 5).

Until the 1960s, immunity was only considered in the context of an antibody response. Jerne in 1955 (6) proposed small amounts of antibody to antigen circulates in blood and when bound to antigen, the complex induces cells to produce more antibody to the antigen. Burnet modified Jerne’s theory to hypothesise that cells had pre-formed antibody to only one antigen, and that antigen activated these clones to produce antibody to the antigen (7, 8). That B cells produce only one specific antibody, was demonstrated by Burnet’s student Gus Nossal, together with Nobel Laureate Joshua Lederberg, in experiments using an assay of flagella immobilization after immunization with two bacteria with different flagella antigens (9, 10).

At that time, the fate of lymphocytes was unknown. There were two hypotheses; one that lymphocytes could differentiate into many different cell types, and the second that they were terminally differentiated cells that could not divide (11). The central role of lymphocytes in immunity was not appreciated until Gowan’s work on thoracic duct lymphocytes (TDL) in the early 1960s (12, 13). Thus, the clonal deletion theory was accepted before the role of lymphocytes in immunity was known or recognized.

### Transplant Rejection

The quest for transplant tolerance arose from work to examine if allogeneic tissue could be accepted, so making tissue transplantation clinically possible. To understand why skin grafts failed in burns patients (14), Peter Medawar went from bedside to bench. He observed rabbits that had rejected a skin graft, had accelerated second-set rejection of a subsequent graft from the same donor but not of third party grafts (15). This suggested rejection was an immune process. Prior studies on transplanted tissue had not supported an immune mediated response (16), but others did (17). The inflammation causing loss of a skin graft was associated with a lymphocyte, not a granulocyte infiltration (18). At that time antibodies, not lymphocytes, were considered the mediator of rejection (19).

Medawar’s group was asked by the Animal Breeding Research Organization to perform skin grafts between cattle twins as a means of distinguishing fraternal and identical twins. Fraternal twins accepted the other’s skin grafts but rejected third party grafts (18, 20, 21). The twin calves shared red cells and the possibility they were chimeras was raised, as female twins had male cells (18).

Work of Gorer and Snell, starting in the late 1930s with breeding of congenic strains of mice, identified the Major Histocompatibility Complex (MHC) as the genes that promoted rejection of transplanted tissue and tumours (22–24). Highly inbred strains provided models with known MHC incompatibility, that were used to define the mechanisms of rejection and transplant tolerance.

### Induction of Transplant Tolerance *In Utero* and in Neonates in Murine Models

In 1953 Billingham, Brent and Medawar injected newborn mice with donor cells, and found that later in life these mice accepted specific donor skin grafts and normally rejected third party grafts (25–27). Woodruff replicated these findings in neonatal rats (28, 29). Skin grafts applied to newborn rats were accepted to varying degrees and second skin grafts from the same donor strain were delayed in rejection, as were donor strain thyroid grafts (30).

Transfer of normal unsensitised recipient strain cells to mice with tolerance induced slow rejection of some but not all grafts (31). A second donor strain skin graft often was slowly rejected without affecting the original graft, suggesting some anti-donor immune reactivity was present. It was proposed that there is incomplete clonal deletion (28). Some mice induced to become tolerant at birth developed runt disease (26) and autoimmunity (32) indicating an aberrant interaction of the tolerizing process and immune responses to self. These unexpected findings were not explained at that time. They suggest a loss of autoimmunity control mechanisms.

### Induction of Transplant Tolerance in Developing Chickens

In the early 1950s, skin grafts in chicken eggs and newborn chickens were also studied albeit histocompatibility could not be matched (19). In a small proportion of transplants, skin grafts between newly hatched chickens of different strains had prolonged survival with good feather growth (19, 33, 34). In some chicks, there was delayed loss of feathers from chronic rejection and these had a lymphocytic infiltrate (35). However, chicks with grafts that appeared tolerated rejected a second skin graft from the same donor but usually retained the original graft (36). This suggested graft accommodation and there was no specific systemic transplant tolerance.

Hasek in Czechoslovakia showed parabiosis of chicken eggs suppressed the haemagglutinin response to the paired chicken (37) and a skin graft from the parabiosis partner was accepted (37, 38). This work was published, in a Czech journal of limited circulation, in the same year that Medawar’s group reported neonatal induced tolerance in mice. Hasek interpreted his findings in relation to the Stalinist theories enforced by Lysenko and Michurin, which ignores genetics (39). When aware of Billingham, Brent and Medawar’s findings in mice, Hasek re-interpreted his experiments significance for transplant tolerance (40).

In birds, embryonic cross transfusion of RBC alone induced graft survival as did bone marrow cells (37). Embryonic cross-transfusion was most effective at 12-18 days post fertilization, suggesting early exposure to alloantigen is required (41).

In 1957, Simonsen reported leukocytes induce reactions on chick membranes (42) as reported by Murphy in 1916 (43). Transfer of white cells to the embryo results in non-antigen specific delay in rejection of skin, as the injected cells induce splenomegaly in a GVH reaction (34). Cross transfusion with blood from chicks of the same strain as the donor, but not the actual donor, prolongs donor skin graft survival, showing the pre-treatment with allogeneic cells is not always alloantigen specific (44). The non-alloantigen specific immune depletion is due to GVH response mediated by transferred immunocompetent cells.

Studies with chicken eggs replicated those with murine models. Both can induce alloantigen specific tolerance, but there also can be non-specific immunosuppression due to GVH response mediated by transferred cells.

In 1960, Medawar and Burnet shared the Nobel Prize for Medicine and Physiology “*For discovery of acquired immunological tolerance”*. The key observations leading to the concept of clonal deletion are listed in **Figure 1** and **Supplementary Table 1**. Although, at that time there was evidence for clones of B cells, the thymus was considered irrelevant. The data on antigen specific tolerance in neonates was consistent with immune ignorance that could be due to clonal deletion or specific inhibitory mechanisms.

### Induction of Transplant Tolerance *In Utero* and in Neonates in Large Mammals

The experiment of nature in Freemartin cattle demonstrates that Medawar type tolerance induction could occur in large mammals. To examine if Medawar-like transplant tolerance can be induced in larger animals, MHC incompatible bone marrow depleted of T cells were infused *in utero* to miniature swine. Induction of tolerance was evident by induction of chimerism, low reactivity of lymphocytes to donor alloantigen and acceptance of a donor kidney allograft (45, 46).

Combined, these studies support the notion that donor alloantigen during embryonic development induced a state of immune hypo-responsiveness to tissue transplant from the same donor strain. This was interpreted as clonal deletion.

### Evidence That Exposure to an Alloantigen *In Utero* or at Birth Does Not Always Induce Clonal Deletion

The mechanism of neonatal tolerance induction is not universal and the reason for the failure to induce tolerance was not considered. Not all strain combinations are susceptible to neonatal transplant tolerance induction (47) and this is due to MHC and non-MHC genes (48). In some animals, second donor strain grafts were rejected, albeit often slowly, showing tolerance and therefore clonal deletion was incomplete. Many studies at the time indicated the process of transplant tolerance is not simply clonal deletion.

If transplant tolerance was solely due to clonal deletion, infusion of normal naïve immune cells would cause allograft rejection. TDL effect rejection of long surviving skin grafts on tolerant animals (49). Transferred syngeneic lymphocytes proliferate in tolerant hosts but later host cells produced by the thymus mediated anti-donor reactivity (50, 51). Transfer of host strain naïve lymphoid cells do not always break tolerance, even when large numbers of cells are transferred (31). Parabiosis of an animal with long-term transplant tolerance with a naïve host does not always break tolerance (52), but in other studies tolerance is broken (31).

### Role of Donor Haemopoietic Chimerism in Maintenance of Transplant Tolerance

Persistence of neonatally induced tolerance requires maintenance of lymphoid chimerism (53, 54) including in the thymus (55). The most potent cells for inducing neonatal tolerance are bone marrow (31), although cells from kidney, testes and spleen can also induce tolerance (25). Chimeric cells enter the thymus where they tolerise T cells (55). A skin graft to a neonate can also induce tolerance (30). Persistence of tolerizing antigens is required to maintain tolerance, as treatment with allo-antisera to deplete chimeric cells abolishes tolerance (56, 57). Transfer of neonatal tolerance to irradiated syngeneic hosts, requires transfer of chimeric cells (53).

### The Response of Donor Cells Against Recipient- Graft Versus Host Assays

A GVH response by lymphoid cells is usually by transfer to a host that will not react against the transferred cells (42), usually a F1 hybrid of donor x recipient. Lymph node cells and blood cells (26), as well as small TDL mediate GVH (58), described at that time as runt disease (12). TDL also induces runt disease in rats (59, 64). The small lymphocytes transform into large pyroninophilic cells that divide (12). These cells were similar to cells that may produce antibody described by Fagreus (60) and lymph node and spleen cells induced by a skin homograft (61).

Injection of parental strain lymphoid cells into an F1 host, particularly if the host was irradiated, induce a similar reaction (62, 63). Cells from adult homologous lymphoid tissues induce runt disease in embryo chickens (42), newborn mice (26) and newborn rats (64), suggesting lymphoid cells mediate this GVH reaction (26).

Testing of peripheral lymphoid cells from animals with neonatal tolerance in GVH assays (65) and in mixed lymphocyte cultures (MLC) (66) showed lack of reactivity to specific donor alloantigen but have normal response to third party alloantigen. These studies were interpreted to support the clonal deletion hypothesis for neonatal tolerance.

### The Role of Lymphocytes in Transplant Tolerance

In 1954 Lord Florey stated “nothing of importance is known regarding the potentialities of lymphocytes other than that they move and that they reproduce themselves” (67). This reflected the conclusion of doctoral studies by Jean Medawar, wife of Peter Medawar, who was a student in Florey’s department. She had cultured lymphocytes from TDL and show they did not spontaneously differentiate (11). In the 1950’s Gowans, another member of Florey’s department, showed thoracic duct lymphocytes in TDL recirculate from blood into lymphoid tissue and then back to lymph (68, 69). Later, Gowans showed small lymphocytes initiate immune responses (13, 58), develop into antibody producing cells (70), promote rejection of allografts (49) and GVH responses (12). He showed TDL include both T and B cells (71).

### B Cells

In the mid 1950s, it was shown that bursectomy in chickens impairs antibody production (72) and reduces lymphocyte numbers, but has no effect on rejection of skin allografts (73). In birds, the Bursa of Fabricius was considered similar to the thymus, in that it was a lymphoid organ present in early life that atrophies (74). At that time lymphocytes and the thymus had no known immune function and adult thymectomy had little effect on antibody production (75–77). All immune responses were attributed to antibody, including graft rejection.

Attempts to accelerate graft rejection with antigraft antibody are unsuccessful, whereas sensitised lymphoid cells transfer alloantigen specific rejection (78). In other studies, the presence of anti-donor antibodies delays rejection and enhances survival of the graft, inducing a form of tolerance (77, 79, 80). Preformed alloantibody lead to hyper-acute rejection in man (81), sheep (82) and rats (83), however. The role of B cells in clonal deletion and in the mediation of transplant tolerance is beyond the scope of this review. The central rejection mechanism is a T cell response, both CD4^+^ and CD8^+^ T cells (84).

### The Thymus

In 1961, Miller reported that mice thymectomized within 16 hours of birth were lymphopenic in blood and lymphoid organs, with deficiency in germinal centres and plasma cells (85–87). The neonatally thymectomized mice, accept allogeneic skin grafts from 41 to over 100 days, whereas sham thymectomized and normal mice reject all grafts in 10-11 days. The animals with surviving skin grafts were described as tolerant, but many died of runt disease. At that time runt disease was considered due to infection and was not induced in specific pathogen free mice (88). Runt disease resembles GVHD (89) and a form of autoimmunity seen in FoxpP3 deficient mice (88).

In chickens, thymectomy of neonates, led to an inability to reject a skin allograft but preserved antibody responses (8). Neonatally thymectomized rats (90) and nude mice (91–94) also do not reject allografts. Neonatally thymectomized mice that were grafted with a host strain thymus, and mice thymectomized 5 days after birth rejected skin grafts (87). The thymus is required for recovery of lymphocytes after whole body irradiation (95). Involution of the thymus increases susceptibility to autoimmunity (96), suggesting the thymus plays a key role with aging in maintaining immunity.

Burnet and Jerne immediately predicated that the thymus would be a site of clonal deletion of self-reactive cells (97, 98), which turned out to be true (99–101). On the other hand, after Miller described the effects of neonatal thymectomy (86), Sir Peter Medawar stated in 1962; *“we shall come to regard the presence of lymphocytes in the thymus as an evolutionary accident of no very great significance”* (102).

The fact that T cells, like B cells were clonal was established by the identification of T cells that respond to a specific alloantigen (103) and that sensitized hosts have memory T cells for specific sensitizing alloantigen (104). The cloning of an immunoglobulin like molecule as an antigen specific T cell receptor (105, 106) and the generation of T cell clones by repeated stimulation with antigen, reviewed (107), confirmed there were antigen specific T cell clones. The finding that T cells identify antigen presented by MHC molecules (108), further confirmed T cells were antigen specific.

### Clonal Deletion in the Thymus

The developing CD4^+^CD8^+^T cells in thymus undergo a complex selection process. This has been extensively investigated over the last 60 years as set out in other reviews (109). This review will be limited to and focus on the thymus and T cells role in transplant tolerance.

The majority of thymocytes have no affinity for MHC and by neglect die by apoptosis (110). Thymocytes with strong affinity to self MHC, also die by over activation (110). In this step, APC activate thymocytes that recognize host and they are deleted by apoptosis (101).

After surviving in the thymic cortex, thymocytes enter the medulla where they contact autoantigens. Here, self-reactive T effector lineage cells are deleted and FoxP3^+^Treg lineage that recognize autoantigen survive (111). T cell anergy to antigen requires continued exposure to antigen (112).

The AIRE (autoimmune regulator) molecule plays a major role in deletion of autoreactive cells and promotion of auto-antigen protective Treg, as reviewed (113). AIRE is expressed by thymic epithelial cells located in the medulla of the thymus. These thymic epithelial cells also express class II MHC and CD80. Expression of AIRE, Class II MHC and CD80 on thymic epithelial cells can be observed in day 14-15 mice embryos.

AIRE promotes promiscuous gene expression by thymic epithelial cells, which includes hundreds of genes whose expression is normally restricted to peripheral specialized tissues. Effector T cells with TCR recognizing these autoantigens expressed by Class II MHC on thymic epithelial cells, causes their deletion and central tolerance. Similarly host dendritic cells in thymic medulla, also promote deletion or anergy in thymocytes that recognize self- antigen, to prevent autoimmunity. Thymic epithelial cells are more tolerogenic for CD4^+^T cells than CD8^+^T cells (100).

Donor cells given to induce neonatal tolerance can enter thymic medulla of the host and promote central tolerance by induction of anergy or apoptosis of T cells recognizing the donor alloantigen. In this latter process, donor alloantigen selects for survival of CD4^+^CD25^+^FoxP3^+^Treg, discussed further below. These mechanisms are of relevance to tolerance models where there is chimerism and the thymus is essential (114).

Probably due to murine studies with neonatal thymectomy and the limited consequences of thymectomy in adults, the role of the thymus during life has been underappreciated. During life, the thymus continues to produce naïve T cells, and presumably naïve CD4^+^CD25^+^FoxP3^+^Treg cells (115). After deletion of peripheral T cells by irradiation, chemotherapy as in bone marrow transplantation (116) or HIV infection (117), the peripheral T cell pool is re-established by expansion of remaining T cells in the periphery and later in a delayed fashion by generation of naïve T cells in the thymus (116). IL-7 in thymus promotes production of naive T cells that are exported to the periphery. These cells protect against infection and malignancy, as well as autoimmunity (118).

The thymus by deleting new alloreactive naïve T cells and selecting alloreactive Treg probably contributes to tolerance induction in adults as well as *in utero* and newborn.

### T Regulatory Cells and the Thymus

Thymocytes are prone to develop to Treg (119). Human babies produce CD4^+^CD25^+^Treg at 13 weeks of gestation (120). Thymectomy in the first month of life, usually for cardiac surgery, later in life results in a higher rate of autoantibodies (121) and a reduced naïve T cell pool (122). Children thymectomized in the first year of life, have reduced numbers of T cells, CD4^+^ and CD8^+^T cells and CD31^+^T cells, with a reduced diversity of their TCR repertoire throughout life (123). CD31 is a marker of T cells recently exported from the thymus (123).

In contradiction to neonatal thymectomy depleting immunity, neonatal thymectomy in mice at day 3, not day 0, resulted in autoantibody production (124–127) and a variety of autoimmune diseases. The organ attacked is determined by host genetic factors (94, 128–130). In mice thymectomized as neonates, autoimmunity is prevented by a thymus graft or injection of naïve adult thymocytes or peripheral lymphocytes (131). Neonatal thymectomy of rats also results in development of autoimmunity (132).

Adult thymectomy and whole body irradiation induces thyroiditis in rats (133), that can be prevented by transfer of normal lymphocytes (134). Rat thymocytes depress autoantibody responses (135). Loss of Treg is considered the cause of experimental autoimmune gastritis (128).

**Figure 2:**
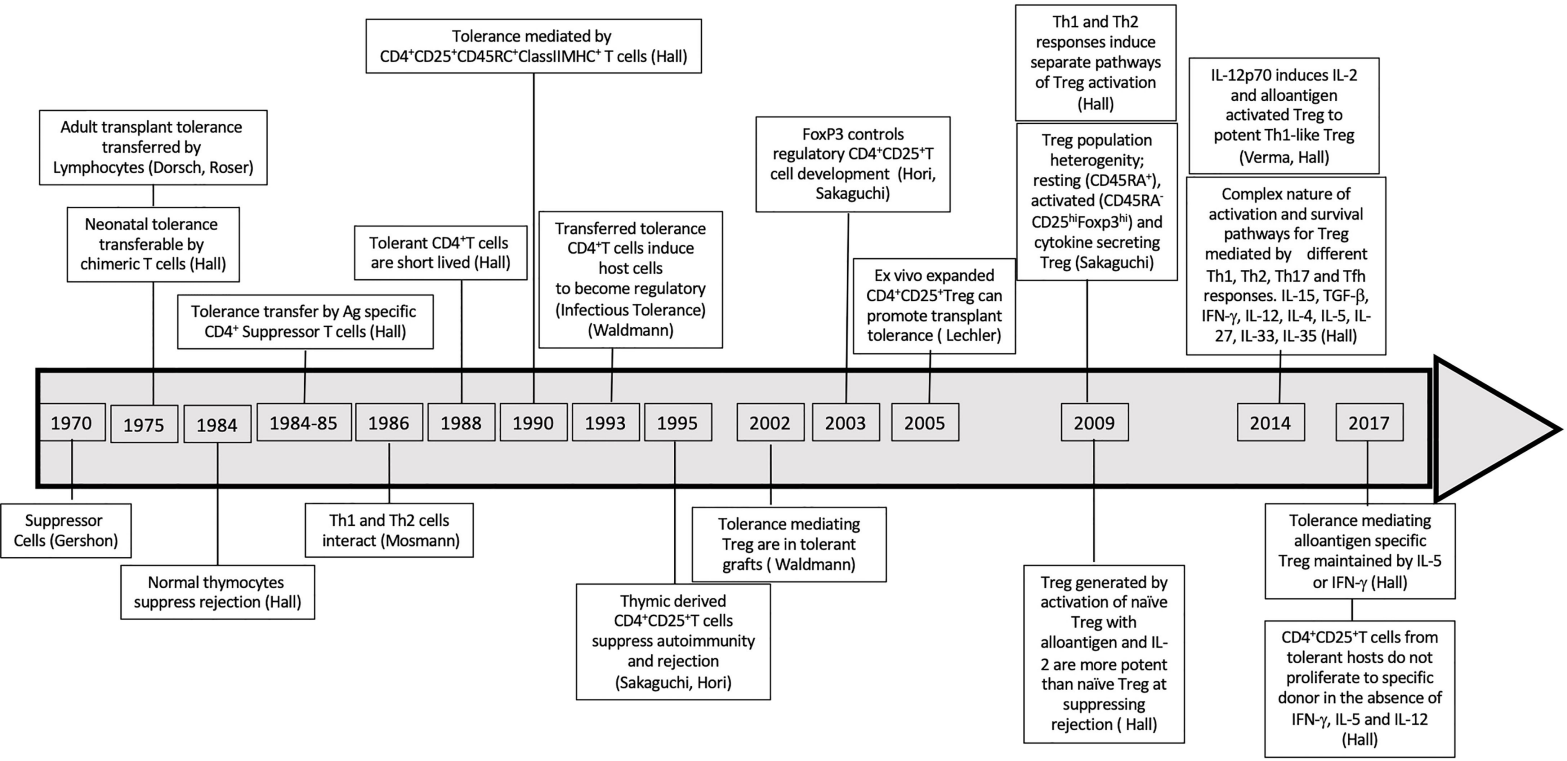
Discoveries Timeline related to suppressor regulatory T cells. Timeline of major discoveries that led to recognition of role of T regulatory cells in tolerance specially in antigen specific tolerance.

In the 1979-80’s, CD8^+^T cytotoxic cells were considered the main mediators of rejection (136) but also included CD8^+^I-J^+^ suppressor T cells. The first reports of suppressor T cells documented their inhibition of B cell responses (137–141). Tissue specific suppressor cells also were shown to protect against autoimmunity (142). Treatment with anti-donor I-J, but not anti-host I-J, broke neonatal tolerance (143). At that time, I-J was considered a marker of CD8^+^T suppressor cells (144), until the gene for I-J was not found (145). This error in phenotyping, led to a decade or more delay in the study of regulatory T cells. Later work on adult models of transplant tolerance, led to the rediscovery of suppressor/regulatory cells, which were CD4^+^T cells not CD8^+^T cells as a major immune cell (146). The key points related to discovery of Treg are summarized in **Figure 2** and **Supplementary Table 2**.

In a mouse model of oophoritis, induced by thymectomy 2-4 days after birth, Ly1^+^ T helper cells prevent autoimmunity (147). Ly1^+^T cells from normal animals were shown to prevent onset of autoimmunity (148). Ly1 is a marker of non CD8 cells, of the helper lineage, now better identified by expression of CD4.

At that time, in 1985, we described that adult transplant tolerance was maintained by CD4^+^T cells, not CD8^+^T cells (149). In 1990, we reported that CD4^+^CD25^+^T cells mediate transplant tolerance (150). This was the first description of a regulatory function of CD4^+^CD25^+^ T cells. We also showed CD8^+^T cells played no role in maintaining transplant tolerance (150). Later in 1995, the Sakaguchis used our finding to show CD4^+^CD25^+^T cells prevented onset of autoimmunity in day 3 thymectomized mice (151).

CD4^+^CD25^+^Treg express the transcription factor FoxP3, which distinguishes them from effector lineage cells (152). During development of thymus, production of FoxP3^+^Treg is delayed compared to production of effector lineage CD4^+^T cells (153). CD4^+^CD25^+^FoxP3^+^T cells control effector CD4^+^ and CD8^+^T cells to prevent induction of autoimmunity (154). Neonatal thymectomy reduces CD4^+^CD25^+^FoxP3^+^Treg that prevent autoimmunity (155–159).

CD4^+^CD25^+^FoxP3^+^T cells that enter thymic medulla contact thymic epithelial cells and dendritic cells that express host autoantigens induced to be expressed by AIRE. This contact of CD4^+^CD25^+^FoxP3^+^T cells with auto-antigen in the thymic medulla promotes their expansion and survival (160, 161). Treg with specificity for an autoantigen prevent autoimmunity.

There is limited information on the role of CD4^+^CD25^+^Foxp3^+^Treg in neonatally induced tolerance. The output of Treg from thymus in the neonatal period, makes it possible that Treg specific for the allogeneic cells are activated alongside CD4^+^CD25^+^Foxp3^+^Treg that prevent autoimmunity.

### Assays of Clonal Deletion of T Cells

Assays of T cell alloreactivity include quantitative GVH (162, 163), MLC (164, 165) and cell mediated lympholysis assays (CML) (166). CD4^+^T cells responding to Class II MHC are assayed in GVH (65, 167) and MLC (168). There is a weak response to Class I MHC (169), which is assayed by generation of CD8^+^T cells to CML in MLC.

Tolerant hosts have reduced frequency of alloreactive T helper cells (170–173) and it is loss of this response, rather than CML that is associated with neonatal tolerance (174). On the other hand, in one study 75% of lymphocytes from animals with neonatal tolerance, responded to donor class II MHC and produced IL-2 (175). Tolerant class II MHC reactive cells on activation *in vitro* produce IL-2, IFNγ, IL-4 and IL-5 (176). In one neonatal tolerance model, lymphoid cells could mediate GVH to specific donor, suggesting incomplete clonal deletion (177). *In vitro* class II MHC responsive tolerant cells undergo apoptosis when re-exposed to donor alloantigen (178).

Cytotoxic T cells (CTL) precursors to specific donor assessed in limiting dilution assays are reduced in neonatal tolerance which is considered due to clonal deletion (32, 170, 171, 179). Donor alloantigen reactive cells are not active in neonatal tolerance, but reactivity to third party alloantigen is retained (180). Lymphoid cells from animals with neonatal tolerance are less cytotoxic to donor cells consistent with clonal deletion (166, 172, 179). However, complete clonal deletion of cells reactive to donor, in some mice strain combinations does not result in transplant tolerance (181). Absence of MLC and CML responses did not predict the induction of tolerance to an allograft (182).

Not all studies show clonal deletion in neonatal transplant tolerance. Cytotoxic T cells effective against Class I MHC are generated in MLC of lymphocytes from tolerant hosts (103, 183), but have reduced function compared to normal cells (184). Other studies showed lymphoid cells from tolerant hosts were not deleted, and were either anergic or suppressed (146). Inhibitory cells or factors were not found in tolerant hosts (185, 186).

### Assay of Tolerant T Cells in Rejection Models

TDL and T cells mediate rejection in whole body irradiated host (187–189), showing antibody and B cells are not essential to the rejection response. Deletion of clones of T cells reactive to donor strain, by passage from blood to lymph in a donor strain host, do not effect rejection but also do not induce tolerance, as recovering host lymphoid cells mount a rejection response (190).

In contrast, TDL from rats with neonatal tolerance do not effect rejection of donor strain skin grafts on irradiated rats, but effect rejection of third party grafts (191). Recirculating T cells from a tolerant host, on adoptive transfer to irradiated hosts, suppresses skin graft rejection (192). This transfer of tolerance is dependent on chimeric donor strain T cells (53, 193). Deletion of chimeric cells breaks tolerance (194). Further adoptive transfer of tolerance requires a donor suppressor T cell (53, 191). Treatment of cells from tolerant hosts with anti-donor sera, removes their ability to transfer tolerance to an adoptive host (53). Thus, the tolerant state depends on the chimeric donor strain cells.

On the other hand, chimerism persists in animals that are not tolerant, and application of the donor skin results in expansion of these chimeric cells, even though the graft is rejected (195).

After neonatal tolerance is broken by transfer of naïve cells, the transferred cells contain all the alloreactivity (51). Later, host thymus derived cells develop and have donor reactivity (50). In these experiments, chimeric donor strain lymphoid cells are lost and cannot promote clonal deletion in the thymus.

The variable results related to GVH, MLC and CML assays, together with the failure of normal cells to effect rejection in tolerant hosts, suggests clonal deletion is not the sole or the essential mechanism for induction and maintenance of transplant tolerance after injection of donor cells in the neonatal period.

### Attempts to Induce Medawar Type Transplant Tolerance in Adults

These pre-clinical and clinical models deplete hosts of peripheral lymphoid cells by irradiation and/or myeloablation and transfer donor lympho-haemopoietic cells to try and establish chimerism. The level of chimerism in these models is greater (up to 80%) (197) than in neonatal transplant tolerance (196, 197) where chimerism is only a few percent of peripheral blood and lymphoid cells (53, 54, 193). The presence of mixed chimerism in blood, thymus and bone marrow indicates donor allografts will be tolerated (198).

These protocols required very high doses of irradiation to allow establishment of chimerism and were too toxic for use in humans. This led to assessment of a variety of immunosuppressive protocols to induce bone marrow chimerism (199).

To reduce the side-effects of whole-body irradiation, total lymphoid irradiation (TLI) is used. TLI targets lymphoid tissues including thymus and spleen and minimizes irradiation of non-lymphoid tissues including skull, lungs, limbs and pelvis (200, 201). TLI given before transplant induces tolerance to organ grafts in rats (200), dogs, non-human primates and humans (201, 202).

In rats, infusion of donor bone marrow cells post-transplant induces chimerism, and the rate of chimerism is high in animals where the thymus was protected from irradiation (203). GVH is not induced by the infused allogeneic cells. In this study, early post-transplant there was non-alloantigen specific hypo responsiveness of host lymphoid cells, which after months became alloantigen specific (203). These host accepted long-term fully allogeneic heart allografts. Post TLI transplant tolerance is maintained by a combination of clonal deletion and suppression (197).

Non-myeloablative regime of non-lethal doses of irradiation, thymic irradiation and T cell depletion, can be used to establish myeloid chimerism and the potential of transplant tolerance (204). These chimeric models of transplant tolerance can be due to central and peripheral tolerance.

To overcome the need to give TLI pre-transplant, TLI was tested by use of anti-lymphocyte antibodies and conventional immunosuppression, which is tapered once TLI was administered. In a high responder rat strain, a combination of anti-CD3 mAb and TLI induced tolerance to fully allogeneic heart grafts and this synergized with donor blood transfusion (205). In this model chimerism was not established (205).

Two groups, one at Stanford and the other Medeor Therapeutics are using post-transplant TLI and anti-thymocyte globulin in renal transplant recipients (206). Early post-transplant, these patients receive some conventional immunosuppression, which is later withdrawn. In HLA matched related donor transplant, infusion of CD34^+^ cells and some T cells, has established chimerism in a large proportion and many are off immunosuppressive treatment. There were no serious infections or engraftment syndromes which are a form of GVH. Some patients required long-term immunosuppression. With HLA incompatible grafts, there was an engraftment syndrome in some patients, and chimerism was lost (206).

### Induction of Bone Marrow Transplants to Induce Transplant Tolerance

Several other protocols have been described, with success. These protocols are discussed in detail in a recent review and will not be described here (206). The details of these protocols and the immunological mechanisms operating are incompletely understood and are beyond the scope of this review.

## Induction of Transplant Tolerance in the Adults, Specific Unresponsiveness Without Clonal Deletion

There are several methods of inducing “Operational Tolerance” in adult animals, and many do not induce clonal deletion. These models and the non-clonal deletion mechanisms by which they are induced and maintained will be reviewed. Key mechanisms of induction and maintenance of transplant tolerance in adults are listed in **Table 1**.

**Table 1:** Immune Mechanisms described in Transplantation Tolerance.

Mechanism	
Clonal Deletion	CD4^+^ cells
	CD8^+^ T cells
	B cells
Clonal Exhaustion	Apoptosis
Clonal Anergy	Systemic donor hypo-reactivity
Specific unresponsiveness	CD4^+^ cells transfer from tolerant hosts
Regulatory T cells	Naïve Treg
	Activated Treg Ts1, Ts 2, Highly potent Th1-like Th2 like
Chimeric Donor Derived haemopoietic and lymphoid cells	Regulatory
	Effect Clonal silencing
Graft Factors	Alloantigen mass
	Inhibitory factors secretion
Donor Dendritic cells	Depletion/graft adaptation
	Stimulation of regulatory cells
	Failure to stimulate effector cells
Immune ignorance	–
Antibodies to Class II MHC	Blocks CD4^+^T cell activation and effectors
Excessive Immune activation	Dependent on grafts antigen presenting cells

Three broad groups of specific unresponsiveness induced in adult animals where there is no “Clonal Deletion “will be reviewed

### Spontaneous Acceptance of a Directly Vascularized Organ Allograft Without Immunosuppression Induces Specific Unresponsiveness

The best example is allogeneic liver allografts, which in some hosts are accepted without immunosuppression, reviewed (207, 208). This was first observed with liver transplants in pigs (209), but also occurs in rats (211) and mice (210). In rats, such tolerance is only induced in low responder strains whereas in mice liver transplants induce tolerance in nearly all strain combinations. In miniature swine thymectomy reduces the rate of tolerance induction to liver allografts (88).

In rats, liver allografts rapidly induce systemic donor hypo-reactivity (211) and reverse rejection of other donor strain organs (212). There is partial clonal deletion in peripheral lymphocytes (213) including specific donor memory lymphocytes (214).

Liver transplant tolerance induction depends upon passenger leukocytes in the liver graft (215, 216). Immune activation by donor leukocytes in the graft is a major mechanism (217), that leads to clonal exhaustion (218). Compared to heart allografts, there is more rapid migration of passenger leukocytes to spleen and lymphoid tissue with a more rapid activation of T cells (219). There is activation of Th1 responses with induction of IL-2 and IFN-γ (220). Reduction in Th1 response by administration of corticosteroids (221) or the Th2 cytokine IL-4 (222) prevents development of tolerance and promotes liver allograft rejection. Paradoxically, prior treatment of donor with rIL-4 increases macrophages in the donor liver and induces tolerance to livers in strain combinations where liver allografts are not spontaneously accepted (217).

Passenger leukocytes transplanted within the liver allograft can mount a GVH response and provide a source of donor cells (223). Micro chimerism of donor lympho-haemopoietic cells occurs and promotes tolerance (223, 224). Whether this GVH leads to further clonal deletion is unclear. The thymus is essential for stopping GVH in the liver graft but is not required long-term (225).

The mass of the liver protects it from rejection. Activation of T cells by hepatocytes, rather than antigen presenting cells, leads to incomplete activation and rapid loss of function (208). High alloantigen expression in the liver exhausts alloantigen reactive CD8^+^T cells (226). Direct contact of alloreactive T cells with liver cells, through fenestrations in the endothelium of hepatics sinusoids, results in their deletion or exhaustion (227). Deletion may also be related to the massive activation of alloreactive T cells (220), which may become anergic or be deleted by apoptosis of T cells (219, 220, 228), including alloreactive T cells (229). Sensitized T cells are deleted in the periphery (214).

In rats, transplantation of a liver immediately stops rejection of a heart graft from the same donor, demonstrating a systemic effect, which may include secretion of MHC molecules from the liver (230, 231) or other immunosuppressive molecules (232, 233).

T suppressor cells have been implicated in tolerance to liver allografts (234). There is limited evidence that FoxP3^+^T cells mediate tolerance to a liver allograft (235, 236). CD4^+^CD25^+^FoxP3^+^T cells are present in rejecting and tolerated liver allografts (210). Therapy with FoxP3^+^Treg has been trialled (237, 238). A combination of donor dendritic cells and CD4^+^CD25^+^Treg is more effective at inducing tolerance in a strain that does not spontaneously accept liver grafts (239). In mice, pre-treatment with anti-CD25 mAb prevents tolerance induction, increases the anti-donor T cells response and reduces apoptosis (235, 240).

Current clinical trials of immunosuppression withdrawal from liver allograft recipients have recently been summarized (206). Operational tolerance occurs in patients with liver allografts.

In some mice strain combinations, kidney allografts are spontaneously accepted, and tolerance is induced. This is associated with induction of FoxP3^+^T cells, not Th1 cell activation (241). With rat kidney allografts, administration of donor leukocytes at the time of transplantation induces donor specific transplant tolerance (242). This increases T cells activation and induction of IL-2 and IFN-γ in the allograft associated with the infusion of donor leukocytes, suggesting overactivation, as occurs with liver allografts, induces tolerance (242, 243).

### Transplant Tolerance With Specific Unresponsiveness Without Clonal Deletion in Large Animals With Kidney and Heart Allografts

In studies with inbred miniature swine, a single or double class I MHC incompatible kidney or heart allograft treated with a short course of cyclosporine A (CSA) (244) or tacrolimus (245) therapy develop a form of tolerance. A large proportion of these animals develop tolerance, with no anti-donor antibodies, variable CML to donor, and most accepted a second donor allograft without immunosuppression. In the swine kidney allograft model, 12 days of high dose CSA induces tolerance and the to be tolerated grafts have a cellular infiltrate not dissimilar to rejection in the untreated kidney allografts, with induction of inflammatory cytokines in both tolerated grafts and rejecting grafts (246). This infiltrate spontaneously resolves (247). This suggests the tolerance is central, and not due to graft adaptation (244, 245). Donor antigen presenting cells in the second transplanted graft do not trigger rejection. The maintenance of tolerance requires the presence of the original tolerated renal allograft (248).

Class II mismatched miniature swine kidney grafts with no immunosuppression are rejected but a short course of CSA induces long term graft acceptance (249). These animals with long surviving allografts accept skin and second kidney allograft from the same donor strain indicating tolerance (249). Class II MHC matching is more important in tolerance induction in miniature swine than class I MHC matching (250). For a second test heart graft to be accepted by a swine tolerant to a kidney graft, the second graft must share class II MHC with the original kidney graft (251).

Host thymus is essential for tolerance induction (252) but not-long term maintenance of tolerance (253). Thymectomized miniature swine are resistant to induction of tolerance to cardiac allografts (254–256).

Co-transplantation of thymus and a kidney allograft enhances tolerance induction (244). Vascularized thymus allografts in miniature swine transplanted at the same time as a heart graft, combined with a short course of tacrolimus, induce transplant tolerance (257). Combined thymus/heart grafts have increased survival compared to heart grafts without thymus (258, 259).

In miniature swine, combined heart and kidney allografts are accepted and induce tolerance, whereas single grafts are rejected. This effect of combined heart and kidney allograft is in part due to increased alloantigen load (260). Irradiation of the kidney but not the heart allograft prevents tolerance to both grafts (255).

In pigs, the transfer of the tolerated kidney with cells from the tolerant host induces prolonged survival in a second irradiated host, suggesting the graft and cells from tolerant animals promote tolerance (261). The tolerated kidney allograft when transplanted to a second host, induces tolerance with or without co-transferred tolerant cells, suggesting a peripheral mechanism of tolerance (261). Tolerated kidneys are rejected when retransplanted into a normal host, indicating graft adaptation is not the mechanism of tolerance (262).

Application of donor strain skin to swine tolerant to a kidney allograft induces anti-donor CTL, but the kidney graft is not rejected (263).

Lymphocytes from miniature swine tolerant to a kidney allograft inhibit responses to specific donor but not third party (264). Tolerant hosts have reduced helper and CTL capacity against donor strain (265). Lymphocytes from tolerant hosts, do not generate CML against donor but do to third party (266). CD25^+^ lymphocytes suppress donor specific CML (267).

Prior specific donor blood transfusions increased the rate of induction of tolerance by CSA to heart transplants, suggesting a peripheral mechanism of tolerance induction (268). Prior induction of tolerance by a bone marrow transplant in swine allows acceptance of donor strain kidneys (269).

These studies in miniature swine, performed by a group at the NIH and Massachusetts General Hospital, show specific unresponsiveness to an organ allograft can be induced by methods used in rodent models that are reviewed below. They establish that acceptance of a graft is (i) preceded by a rejection like response that spontaneously resolves, (ii) is facilitated by the thymus, (iii) is alloantigen specific and that peripheral lymphocytes can promote tolerance and are not clonally deleted. The mechanisms of tolerance induction and maintenance may be very similar to those in the rat models, making it possible that such specific unresponsiveness may be induced in all species including man.

### Split Tolerance or Specific Unresponsiveness in Adult Murine Models

In adult rodents, a variety of treatments reduced rejection and induce a state where organ allografts are accepted without ongoing immunosuppression. This phenomenon was called specific unresponsiveness (270, 271) and is a form of “operational tolerance” (272), reviewed (273). These models of tolerance have similarities to the swine models, as described above. Like in the swine models, a rejection response is generated but is insufficient to reject the grafts which survive. It then takes weeks for tolerance to fully mature after exposure to alloantigen. Tolerance is associated with a loss or change in dendritic cells and the development of suppressor cells, which in all cases include CD4^+^T cells (273).

One of the first such models of transplant tolerance was induced by treatment of mice with donor liver cells and anti-lymphocyte serum, which led to acceptance of fully allogeneic skin (274, 275). The acceptance of these allografts requires induction of suppressor cells now known as Treg (276–279). Overtime, there has been increasing acceptance that Treg contribute to this form of tolerance (280, 281) and that alloantigen from the graft can induce host Treg (282).

Other models of specific unresponsiveness, described in the 1960-70s, were passive or active enhancement (283). In these models, there was no attempt to induce chimerism or clonal deletion. In active enhancement, donor peripheral lymphoid cells or haemopoietic cells are given ivi, either at the time of transplant or 7-10 days prior to transplantation. Class II MHC and B cells promote enhanced allograft survival (284). Passive enhancement is induced by injection of alloantibody to the donor stain (79), particularly alloantibody to Class II MHC (77). Kidney and heart allografts are easier to enhance survival of than skin or lung. Sensitization to donor strain alloantigen prevents induction of enhancement of allograft survival (285). Not all host strains can be induced to develop tolerance of an allograft (286, 287).

It is only after weeks that an enhanced allograft induces a state where a second donor strain graft, usually skin, is accepted while third party grafts are rejected (288, 289).

Long surviving grafts can have pathological lesions of rejection (290) but the graft continues to function.

In part, acceptance of enhanced kidney allografts is due to depletion of donor dendritic cells, so that the graft cannot provoke a rejection response when re-transplanted into a naïve recipient strain host (291–294). The loss of alloantigen stimulation to provoke rejection, is not the sole mechanism as second donor strain allografts, with a normal complement of alloantigen presenting cells, are accepted (288, 289).

Treatment with a short-course of CSA (149, 295–300) is more reliable at inducing specific unresponsiveness to allografts than our enhancement protocol (289, 296). The mechanisms of graft acceptance seems to be similar to those in enhancement models (298), although infiltration of grafts by allospecific CTL is impaired (301). Later, other reliable models of specific unresponsiveness were developed, including therapy with antilymphocyte sera (ALS), blood transfusions (302), anti-CD4 mAb (303–306), a combination of anti-CD4 and anti-CD8 mAb (307), anti-CD3 mAb (308, 309) and anti-CD25 mAb (310). The many models for specific unresponsiveness are reviewed elsewhere (273) and include models of transplant tolerance in adult animals (304).

In these models, specific unresponsiveness takes time to develop and is usually not manifest until after 100 days post-transplant (289, 296). It is only after a period of weeks, that second donor strain grafts are delayed in rejection and after time (usually ten weeks) most are accepted (271, 289). Normal rejection of third-party grafts is retained by these hosts at all times post-transplant (271, 289).

Cells from animals with specific unresponsiveness have normal reactivity in MLC (309, 311–313), CML (309, 311, 313–316), and GVH (317–320). There is no experimental evidence for clonal deletion. Animals with enhanced allograft survival make donor specific alloantibody responses (315, 321).

Early attempts to demonstrate suppressor cells in hosts with specific unresponsiveness were unsuccessful (322), but later suppressive T cells were identified (149, 276, 289, 323, 324) and confirmed by others (325–330). The maintenance of suppressor cells is dependent on alloantigen from the graft (331).

The difficulty in measuring suppressor/regulatory cell activity, led us to develop an assay using limited numbers of alloreactivity of cells, and establishing their ability to mediate rejection or transfer specific unresponsiveness in an immunodeficient irradiated host.

## Role and Activation of T Regulatory Cells in Transplant Tolerance

### An Assay to Assess Ability of Lymphocytes to Mediate Rejection or Transfer Specific Unresponsiveness

We developed a model in which different numbers of peripheral lymphocytes capacity to mediate rejection or inhibit rejection of fully allogeneic directly vascularized heart allografts is tested in adoptive hosts whose own lymphocytes had been depleted by whole body irradiation (104, 188). For these studies, DA recipient and PVG heart grafts are used with Lewis rats as third-party donors.

Using cells from naïve animals, we have shown that the most potent are TDL, then lymph node cells and spleen cells (188). Thymocytes and bone marrow cells do not restore rejection (188). Larger numbers of TDL, spleen or lymph node cells mediate faster rejection (188). Enriched recirculating T cells are effective at mediating rejection. Cells from adult thymectomized animals are not impaired and tend to reject faster than cells from non-thymectomized hosts (188, 332). Injecting thymocytes mixed with normal lymph node cells or spleen cells delays rejection (332), suggesting in normal animals thymus cell and peripheral T cells recently produced by thymus inhibit the rejection response (332). In this model, host thymectomy allows transferred cells to mediate faster rejection, suggesting the hosts’ immune reconstitution following irradiation promotes development of tolerance (189).

In this model, enriched CD4^+^T cells mediate rejection whereas CD8^+^T cells and B cells do not restore rejection (189). Dilution of CD4^+^T cells, shows that half a million cells are as effective at restoring rejection as two hundred million cells. This allows studies on tolerant cells to be with very small numbers of naïve CD4^+^ T cells (149, 333). That allows the effects of the suppression by tolerant cells to be assayed, which is not possible in hosts with a redundant effector T Cell population.

Removal of CD25^+^ cells from naïve CD4^+^T cells results in more rapid rejection, consistent with naïve CD4^+^CD25^+^T cells non-alloantigen specific effect on allograft rejection responses (334). Mixing 5x10^6^ CD4^+^CD25^+^T cells from naïve animals with 5x10^6^ unfractionated CD4^+^T cells totally suppresses rejection (334). Tolerance was only induced when the mixture was 1:1 (334). Lower ratios of CD4^+^CD25^+^T cells to effector CD4^+^T cells do not supress rejection and at the normal ratio of 1:10 rejection is not suppressed showing that naïve/resting Treg are weak at suppressing rejection (334). Ratios of 1:1 are impossible to achieve long-term in animals.

Cells from syngeneic donors sensitized to specific donor strain by rejection of skin grafts are more potent (104). Compared to naïve cells, these cells from TDL, lymph node and spleen, accelerate rejection of specific donor allografts but not third party grafts, showing an increase in potency and in alloantigen specific memory T cells (335). These memory T cells do not rapidly recirculate from blood to lymph (104, 336), consistent with what is now known as effector memory T cells. Memory CD4^+^ and CD8^+^T cells mediate rejection, showing sensitized or memory CD8^+^T cells mediate rejection without help from CD4^+^T cells (337).

### Transfer of Specific Unresponsiveness by Lymphocytes

This model of rejection was adapted to the study of specific unresponsiveness. We use DA rats as specific unresponsiveness to PVG heart grafts can be induced by a variety of treatments including passive enhancement (289), CSA treatment (296, 317), anti-CD4 mAb treatment (303, 338) and anti-CD3 mAb treatment (309). In this assay, the relative potency of different cell populations can be examined.

Our studies show peripheral lymphoid cells, especially spleen cells and lymph node cells, but not thymocytes transfer alloantigen specific tolerance (288, 289). B cells and an antibody response is not required to transfer tolerance (297). Enriched T cells populations transfer tolerance to specific donor grafts to an adoptive host (289, 296, 297). Peripheral lymphocyte from tolerant hosts, suppress the ability of naïve peripheral lymphoid cells to restore rejection (289, 296, 297, 339). The tolerant CD4^+^T cells must be at ratios of ≥4:1 to naïve cells. This ratio of specific unresponsive host cells to naïve cells is used in all our subsequent experiments on suppression of rejection. Such ratios of tolerant CD4^+^T cells to host naïve lymphocytes cannot be achieved in normal adoptive host. Thus, tests of transplant tolerance transfer need to use severely immunocompromised hosts, such as those given whole body irradiation, B rats, Rag and SCID mice.

We prepare T cells that recirculate from blood to lymph, by injecting irradiated recipient strain rats with lymph node and spleen cells from rats with specific unresponsiveness. The T cells that recirculate from blood to lymph, do not suppress rejection in a third adoptive host (289). Thus, suppressor T cells from specific unresponsive hosts migrate to peripheral tissue, not secondary lymphoid tissue, and behave like effector memory T cells (104).

### CD4^+^T Cells, Not CD8^+^T Cells, Mediate Specific Unresponsiveness

Examination of the role of CD4^+^T cells and CD8^+^T cells (340) in specific unresponsiveness, produced what is a very surprising result, reviewed (341). That is the CD4^+^T cell fraction transfer specific unresponsiveness, whereas the CD8^+^T cells do not inhibit graft rejection or transfer specific unresponsiveness. A key role of CD4^+^T cells in maintaining unresponsiveness to an allograft is shown in enhancement (319), after CSA treatment (297), anti-CD3 mAb treatment (309) and anti-CD4 mAb treatment (303) mAb. Up until that time suppressor cells were considered to be CD8^+^T cells (cytotoxic/suppressor) not CD4^+^T cells, which were helper/inducer.

Early after transplantation, when there is immunosuppression to induce tolerance, at 8 and 20 days post-transplant, CD4^+^T cells effect rejection (342). It is only after 50 days that tolerance is transferred by CD4^+^T cells (342), consistent with the observation that second donor strain graft are only accepted after 50 days post-transplant (342). With regards to CD8^+^T cells, at 8 and 20 days post-transplant, they effect rejection, much like CD8^+^T cells from controls where no immunosuppression is given (342). CD8^+^T cells at 50 and >75 days do not effect rejection, and do not suppress rejection (342). These studies show that during induction of specific unresponsiveness the hosts CD4^+^ and CD8^+^T cells have capacity to effect rejection and in the case of CD8^+^T cells are activated. With time, the CD4^+^ tolerance mediating cells develop and prevent rejection of a second donor allograft.

Further characterization of the CD4^+^T cells from tolerant hosts, show they cannot suppress specific donor rejection mediated by sensitized CD4^+^T cells, but can suppress rejection mediated by specifically sensitized CD8^+^T cells (150). Depletion of the adoptive host of CD8^+^T cells by thymectomy or treatment with an anti-CD8 mAb demonstrated that CD8^+^T cells are not required to re-establish tolerance in the adoptive host, neither was a thymus in the adoptive host (150). These studies in 1985 were the first to show suppressor/regulatory cells are CD4^+^, not CD8^+^T cells.

In mice with adult induced transplant tolerance, tolerant CD4^+^T cells promote induction of tolerance in host T cells, a phenomenon called “infectious tolerance” (282).

In animals with specific unresponsiveness to an allograft, removal of the allograft 50 days post-transplant results in a loss of tolerance transferring CD4^+^T cells within 8 days and these cells effect rejection in the adoptive host (150). Further, cyclophosphamide treatment of the animals with specific unresponsiveness depletes CD4^+^T cells with the ability to transfer specific unresponsiveness (150). These experiments show that a subpopulation of cells that suppress within tolerant CD4^+^T cells, are rapidly dividing and need alloantigen stimulation. Such activated T cells usually require cytokines to promote their survival and activation. This led us to examine which cytokines could promote their survival and proliferation.

Shortly after our description of a CD4^+^T cell mediated suppression of rejection, Goran Moller in an editorial entitled “Do Suppressor cells Exist?” (145), cited three reasons for doubting the existence of T suppressor cells. First, there was no marker for T suppressor cells to distinguish them from CD8^+^cytotoxic T cells. Second, the gene for the purported marker of suppressor cells “I-J” was not found in the MHC region of mice (343). Third, there was no evidence that the alpha and beta chain of TCR are expressed in suppressor cells, making the existence of antigen specific suppressor T cells impossible.

In the late 1980’s suppressor cells became unfashionable, and many if not most immunologists considered they did not exist and that the apparent suppression described were random artifacts. Suppressor T cells could not be mentioned in polite immunological circles as manifest by Jan Klein (1990) in a preface to his textbook on immunology (344), stated *“I have attempted to find the fundamental truth in immunology and to separate it from hypothesis, regardless of how fashionable they might have been at the time of writing. Consequently, the reader will not find certain topics (such as specific suppressor T cells) discussed at any length, as they are judged not to be a fundamental truth.”* The derision was as blunt as that of Medawar’s dismissal of lymphocytes and T cells as mediators of immune responses such as transplant rejection.

Our work in the mid 1980s shows suppression is mediated by CD4^+^T cells not CD8^+^T cells. These cells transfer alloantigen specific suppression and are not non-specific. Suppression by thymocytes was natural and not antigen specific. In 1990, the role of CD4^+^CD25^+^T cells as inhibitors in transplant tolerance was first described (150). But work on suppressor cells became so unfashionable, its grant funding was cut.

To address the paradox that CD4^+^T cells effect rejection and could also maintain transplant tolerance, we looked for other markers of the suppressor T cell subset.

### Tolerance Promoting CD4^+^T Cells Die Without Specific Alloantigen and Cytokines

We observed in studies to characterize the specificity of suppression by CD4^+^T cells, that culture of CD4^+^T cells with specific donor antigen presenting cells, led to a loss of capacity to suppress rejection of specific donor and a gain in ability to effect rejection (340). This occurred within three days of culture (150, 340). We then cultured the tolerant CD4^+^T cells with specific donor stimulator cells and supernatant from ConA activated splenocytes. This cytokine rich media promoted survival of suppressor CD4^+^T cells, only if specific donor stimulator cells were present.

We found IL-2 partially maintained suppressor function (345) and that depletion of CD25 expressing cells from tolerant CD4^+^T cells removed their capacity to suppress rejection in our adoptive transfer assay (150).

As an anti-idiotypic response was suggested (346, 347), CD4^+^T cells from hosts with specific unresponsiveness were cultured with idiotype of donor alloantigen activated T cells from a naïve host. Even in the presence of supernatant from Con A activated splenocytes, suppressor function of tolerant CD4^+^T cells was lost in culture with idiotype expressing cells (311, 345).

As Con A supernatant is rich in IL-2, we examined if the cells that transfer tolerance expressed the IL-2 receptor, which is now known as CD25. In an attempt to phenotype the CD4^+^T cells that suppress rejection from the CD4^+^T cells that can mediate rejection, we deplete CD25^+^ cells from tolerant CD4^+^T cell. Depletion of CD4^+^CD25^+^T cells, left a population of CD4^+^CD25^-^T cells that mediate rejection of specific donor grafts (150, 348). This work published in 1990 was the first demonstration of CD4^+^CD25^+^T cells as a regulatory or suppressor cell. This observation, discussed with the Sakaguchis, led them in 1995 to report CD4^+^CD25^+^T cells prevent autoimmunity in mice thymectomized in the neonatal period (151). The identification of CD4^+^CD25^+^T cells as suppressor cells slowly led to rehabilitation of the concept of regulation within the immune system.

There is now widespread acceptance that CD4^+^CD25^+^T cells suppress all immune responses. The naïve Treg described by the Sakaguchi’s are very different to the CD4^+^CD25^+^T cells that mediate transplant tolerance. Naïve Treg suppression is not antigen-specific, whereas our tolerant cells transfer donor alloantigen specific suppression.

We also showed tolerance transferring CD4^+^Treg express CD45RC (150), a marker of an activated Treg whereas naïve Treg express CD45RA (349). Tolerance transferring Treg also express Class II MHC, a marker of activated Treg (150). CD45RA^-^(CD45RC^+^), Class II MHC^+^ remain two key markers of activated Treg, that can be used to distinguish them from naïve Treg (349, 350).

### In Our Models of Tolerance, rIL-2 Alone Did Not Sustain Suppressing CD4^+^T Cells That Transfer Tolerance

Although specific transplant tolerance is transferred by CD25 expressing cells, and survival of these cells in culture requires a cytokine rich supernatant from ConA activated lymphocytes, use of recombinant IL-2 alone in culture does not fully sustain the suppressor capacity of these cells (345). This raises the possibility that the alloantigen activated CD4^+^CD25^+^Treg needed cytokines other than IL-2 to promote their proliferation and survival. We tested other cytokines and found key roles for several.

At that time the description of Th1 and Th2 responses (351) resulted in a hypothesis that deviation to Th2 and reduced Th1 responses may explain specific unresponsiveness. We found specific unresponsiveness could be induced by suppression of either Th1 (338) or Th2 (308) responses. Specific alloactivated CD4^+^Th2 cells generated *in vitro*, mediate rejection not tolerance (352–354). Thus, alloantigen specific suppression is not mediated by a switch to a Th2 response, albeit Th2 cytokines such as IL-4 (355, 356) and IL-5 (357, 358) inhibit rejection and promote transplant tolerance induction.

**Figure 3:**
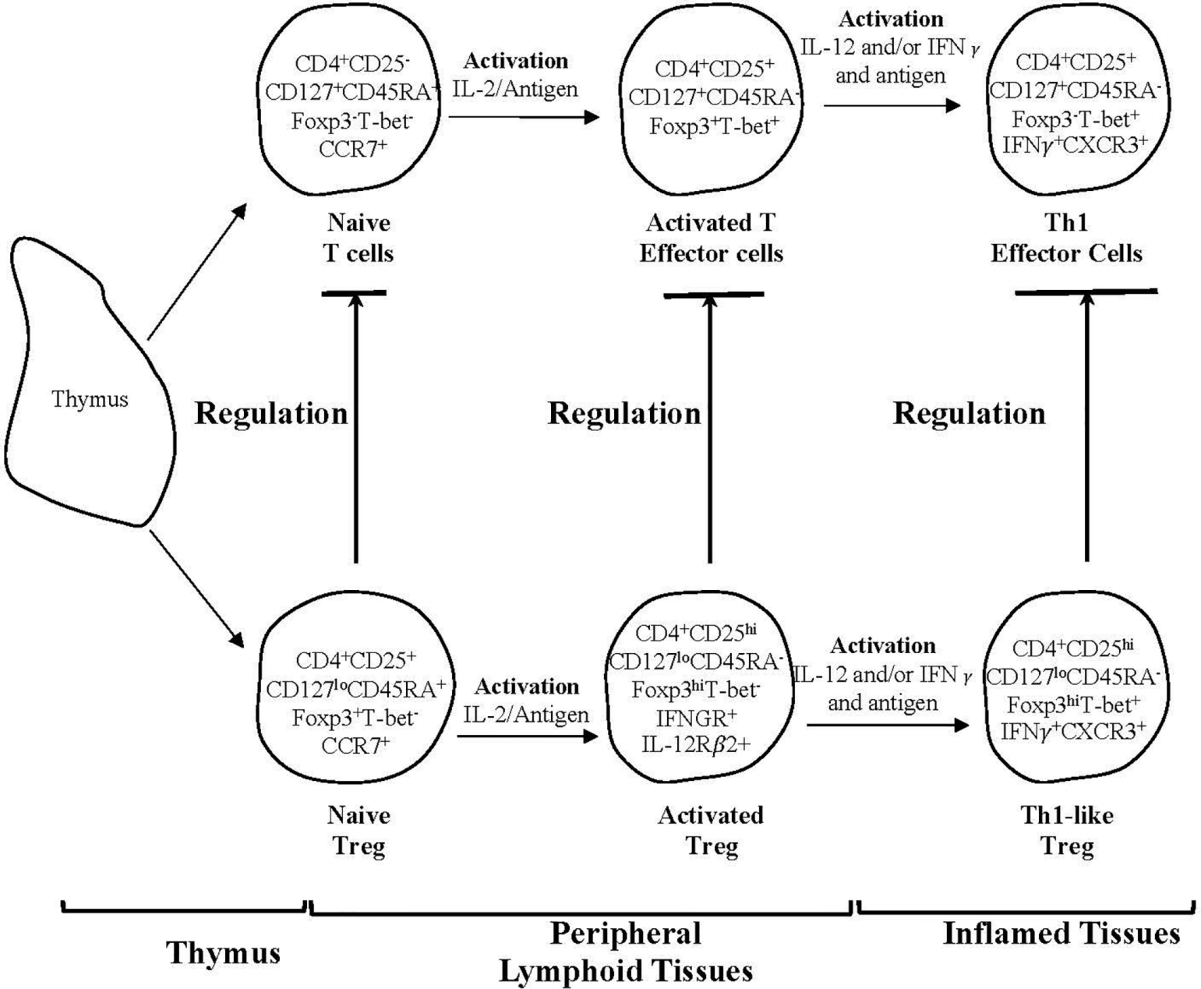
A schematic representation of two subpopulations of CD4^+^T cells produced by the thymus and one of several pathways for their activation by an antigen and cytokines in peripheral lymphoid tissues and sites of inflammation. The activation by an antigen of effector lineage CD4^+^CD25^-^CD127^+^CD45RA^+^Foxp3^-^ cells induces them to produce cytokines that promotes activation of CD25^+^CD127^lo^CD45RA^+^Foxp3^+^Treg that have been activated by antigen. This figure shows the parallel pathways of activation of effector and regulatory CD4^+^T cells, when producing and being activated by Type-1 cytokines. The cytokines produced by the effector cells are required for the full activation of Treg. Both lineages of cells have been produced by thymus and have migrated to peripheral lymphoid tissue. Their subsequently recirculation from lymphoid tissue to blood and back to lymphoid tissue, is promoted by expression of CD62L and CCR7. This recirculation increases their chances of recognizing antigens. In peripheral lymphoid tissue upon recognition of an antigen, both effector and regulatory CD4^+^T cell populations are activated and proliferate. Effector lineage CD4^+^T cells start producing IL-2 and express IL-2R including CD25 (IL-2Ra chain). Naïve resting Treg expand polyclonally. During an immune response naïve/resting CD4^+^CD25^+^CD127^lo^CD45RA^+^Foxp3^+^T-bet^-^CCR7^+^Treg are activated by an antigen and the IL-2 produced by activated T effector cells and are induced to express the receptor for late Th1 cytokines IL-12 and IFN−γ. Naïve CD4^+^CD25^+^CD127^+^CD45RA^+^Foxp3^-^T-bet^-^CCR7^+^T cells also acquire CD25, Foxp3 and T-bet expression but no longer express CD45RA. Transient expression of Foxp3 and CD25 on activated effector T cells blurs the distinction between Treg and effector T cells. In the event of ongoing immune response, activated T effector cells, in the presence of IL-2 and IFN-γ get further activated to express the transcription factor t-bet and the chemokine receptor CXCR3. These activated effector CD4^+^T cells produce IFN-γ, which together with IL-12 further activate Treg to Th1-like Treg (CD4^+^CD25^hi^CD127^lo^CD45RA^-^Foxp3^hi^T-bet^+^IFN-γ^+^ CXCR3^+^). Th1-like Treg express mRNA for Th1 transcription factor T-bet, Th1 cytokine IFN-γ and Th1 chemokine receptor CXCR3. Expression of CXCR3 enables these Treg to migrate to inflamed tissues, where they control immune inflammation as in the graft and promote tolerance. Th-like Treg, such as Th1-like Treg are the mediators of transplant tolerance and are a hundred to a thousand-fold more potent at suppression of rejection than naïve resting Treg. This figure only represents one pathway of activation of Treg and there are others such as Th-2 like Treg promoted by Th2 cells and Type-2 cytokines. The survival of highly activated Treg is dependent on continued antigen stimulation and key cytokines produced by the inflammatory response, IL-2 alone does not sustain these cells and may inhibit them.

Given IL-2 alone does not sustain full suppressor function in CD4^+^T cells from animals with specific unresponsiveness (345), we examined the possible role of other Th1 and Th2 associated cytokines. **Figure 3** shows the parallel pathway of activation of Th1 and Th1-like Treg that we have described.

To do these studies we obtained clones or cloned a variety of rat T cell cytokine producing cell lines. Treating rats with fully allogeneic neonatal heart allografts, we found IL-12p70 (359, 360), IL-4 (356), IL-5 (357) and IL-13 (361) delay rejection, while rIL-2 promotes rejection. To our knowledge no cytokine therapy induces specific unresponsiveness to an allograft, except IL-5 in a chronic rejection model with only Class I MHC incompatibility (358).

The mechanism by which these cytokines delay rejection is unclear. Thus, we examined the effect of various cytokines on CD4^+^CD25^+^Treg in culture with and without alloantigen. IL-13 inhibits macrophage activation, not Th1 cell activation (361) and to date has no reported effect on Treg.

First, we enriched naïve CD4^+^CD25^+^Treg and cultured them with alloantigen or self-stimulator cells. Different cytokines were assayed for their effects on proliferation. Both rIL-2 and rIL-4 induce proliferation to self and alloantigen. Alloantigen alone induced a small proliferative response (312). rIFN-γ, rIL-12, rIL-5, rIL-13, rTGF-β and rIL-10 do not induce proliferation of naïve CD4^+^CD25^+^FoxP3^+^T cells (312).

When CD4^+^CD25^+^FoxP3^+^Treg from animals with specific unresponsiveness were assayed in MLC against self, specific donor and third party, we observed a difference in response to that of naïve CD4^+^CD25^+^FoxP3^+^T cells. Most interesting is that their response to specific donor is at background levels, that is there is no response (312). Their response to third party remains normal (312). This result is consistent with our earlier observation that the ability of CD4^+^T cells from specific unresponsive host was lost within days of culture with specific donor alloantigen in the absence of Con A supernatant (340, 345). Again, rIL-2 and rIL-4 increase proliferation of tolerant CD4^+^CD25^+^T cells to self, specific donor or third party. Three cytokines induce increased proliferation to specific donor but not to self and third party (312). These are rIL-5, IL-12 p70 and IFN-γ whereas TGF-β, rIL-10, rIL-13 do not promote proliferation to specific donor (312).

These studies showed tolerance transferring cells may depend on these cytokines. We tested this by culture of tolerance transferring CD4^+^T cells with specific alloantigen and one of these cytokines. IFNγ (362) and IL-5 (363) sustain their tolerance transferring capacity, whereas cells cultured with rIL-4 cannot transfer tolerance and mediate rejection (352). Further evidence that cytokines other than IL-2 are required to sustain survival and proliferation of tolerance mediating CD4^+^T cells.

### Activation of Naïve CD4^+^CD25^+^FoxP3^+^Treg by Alloantigen and T Cell Cytokines Induces Expression of Other T Cell Cytokine Receptors

These observations led us to examine cytokine receptor expression after naïve CD4^+^CD25^+^FoxP3^+^Treg are cultured with alloantigen and either rIL-2 or rIL-4 (364). This uncovered pathways whereby naïve CD4^+^CD25^+^FoxP3^+^Treg are activated to more potent antigen specific Treg, reviewed (365, 366). Those cultured with rIL-2 and alloantigen or autoantigen are induced to express the receptor for IFN-γ and IL-12 (364, 367), but not the receptor for Th2 cytokines such as IL-5 (364). We call the naïve Treg that had been activated with the Type 1 cytokine IL-2 and express receptors for the Type 1 cytokines, IFNγ and IL-12, Ts1 cells (364, 368). The naïve Treg when activated by the Type 2 cytokine IL-4 and antigens they are induced to express receptor for the Type-2 cytokine IL-5 (364, 369). We call these rIL-4 and alloantigen activated cells Ts2 (364, 369).

In MLC, Ts1 and Ts2 cells suppress responses to specific donor at ratios of 1:32-1:64 (364), whereas naïve CD4^+^CD25^+^T cells only fully suppress MLC at 1:1 -1:2 (168). On adoptive transfer to irradiated hosts restored with 5x10^6^ naïve CD4^+^T cell, Ts1 or Ts2 cells suppress rejection at 1:10, whereas naïve CD4^+^CD25^+^Treg only suppress at 1:1 to effector CD4^+^T cells (334).

Ts1 cells are activated to express CD8 as well as CD4 becoming double positive cells (370). The double positive cells are the cells with the increased potency (370). Further, activated Ts1 cells have increased expression of CD62L (370), suggesting they are programmed to migrate to other peripheral lymphoid tissues, not to the site of inflammation in the graft.

### Cytokines Other Than IL-2 Activate CD4^+^CD25^+^Treg

We next examined if Ts1 cells could be further activated by culture with rIL-12p70 and specific donor alloantigen. In the absence of rIL-2, rIL-12 induces Th1-like Treg (367). These Th1-like Treg suppress in MLC at 1:1000 and are the most potent Treg described. Small numbers of these cells can inhibit allograft rejection in a normal host. Th1-like Treg express T-bet, the Th1 transcription factor, as well as FoxP3, and express IFN-γ but not IL-2 (367).

Ts2 cells can be further activated by rIL-5 in the absence of IL-4, to develop a Th2-like phenotype, expressing the Type 2 transcription factors GATA3 and IRF4, as well as Type 2 cytokines IL-5 (358). They do not express Type 1 cytokines and transcription factors (358).

In man, there is increasing evidence that in parallel with activation of Th1, Th2, Th17, Tfh responses, there is activation of naïve CD4^+^CD25^+^FoxP3^+^Treg to a phenotype similar to the effector lineage (371, 372). That is T-bet and IFNγ with Type-1 cytokines (359) and GATA3, IRF4 and IL-5 with Type-2 cytokines (358).

In humans, CD4^+^CD25^+^FoxP3^+^ Treg can be isolated by their lack of expression of the IL-7 receptor CD127 (373). Focussing on the CD4^+^CD25^+^FoxP3^+^CD127^-^ Treg memory/activated Treg can be distinguished from resting Treg by their low expression of CD45RA (349). Some activated/memory Treg express CXCR3, CCR6 or CCR8, the chemokine receptors respectively expressed by Th1 (371), Th17 (374) and Th2 cells (371). These cells called Th-like Treg respectively express transcription factors T-bet, RORγt and GATA3. CXCR3 promotes cell migration to its ligand CXCL10 expressed at sites of Th1 mediated inflammation (375). CCL20 is induced by IL-17 and produced by Th17 cells, promoting migration to sites of Th17 inflammation (376, 377). Th1 like Treg produce more IFN-γ than other Th-like Treg (372). Th17-like Treg produce more IL17 and Th2-like Treg produce more Th2 cytokines, including IL-4, IL-5 and IL-13 (372). A proportion of activated CD4^+^CD25^hi^FoxP3^hi^CD45RA^-^Treg express both CXCR3 and CCR6 and are Th1/17 like Treg. Th2-like Treg express CCR8, the Th2 chemokine receptor.

CCR4 is expressed by all Th-like Treg and promotes migration to its ligands CCL17 and CCL22 produced by dendritic cells in lymphoid tissues (378). Expression of CCR4 by CD25^+^FoxP3^+^T cells is required for induction of tolerance (88).

This activation of potent Treg is a two-step process that produces effector Treg that can migrate to the site of inflammation by expression of the relevant chemokine receptor, such as CXCR3 on Th1-like Treg. These activated Treg do not migrate from blood to lymph, as we observed in the 1980s (289). In the site of immune attack, they can inhibit effector lineage T cells. This inhibition may include killing effector cells, producing a quasi-clonal deletion in the graft.

Nature of activation and survival pathways for Treg is complex and may involve different Th1, Th2, Th17 and Tfh responses. IL-15 (379), TGF-β (380), IFN-γ (381), IL-12 (360), IL-4 (364) , IL-5 (369), IL-27 (382), IL-33 (383), IL-35 (384).

### Activated Treg in Transplant Tolerance Are Different to Naïve CD4^+^CD25^+^FoxP3^+^Treg

The precise mechanisms that effect suppression are not fully known but include CD39 on Treg producing adenosine (385), IL-35 inducing Treg (386, 387), or consumption of essential amino acids (388). Class II MHC expression may contribute to control of inflammation.

It is the activated Treg that maintain immune tolerance, not the resting naïve Treg described by the Sakaguchis. Such highly activated Treg cells have not been generated *in vitro* as a therapy, as most studies use polyclonal expansion of naïve Treg cultured with rIL-2 with anti-CD3 and anti-CD28 mAb (206, 389). Some naïve Treg cultured with rIL-2 and donor alloantigen have been trialled (390). Therapy with Treg is beyond the scope of this review, however the current limited understanding of the processes that activate alloantigen specific Treg of high potency limits these cells full potential when applied to the clinic. To our knowledge no highly activated Th-like T reg have been trialled in the clinic.

Relevant to the key role of antigen activated, inflammation seeking potent CD4^+^CD25^+^FoxP3^+^Treg, the main features are:

- they suppress rejection at ratios of 1:1000 to effector CD4^+^T cells and are more potent than naïve CD4^+^CD25^+^FoxP3^+^Treg (391).
- they are a small fraction of the CD4^+^CD25^+^FoxP3^+^ T cells population and this population remains <5% of CD4^+^T cells in hosts with transplant tolerance.
- their survival is key to the maintenance of transplant tolerance (392).

In contrast, naïve CD4^+^CD25^+^FoxP3^+^T cells only suppress rejection when ratios of 1:1 are achieved. Such high ratios of naïve CD4^+^CD25^+^T cells has only been achieved with rIL-2/anti-IL-2 complex therapy (393). As homeostatic mechanism prevents Treg exceeding 10% of CD4^+^T cells. Other differences between naïve and antigen activated CD4^+^CD25^+^FoxP3^+^Treg have been summarized elsewhere (341, 366, 394, 395). Naïve Treg are identified as CD4^+^CD25^+^FoxP3^+^CD127^lo^CD45RA^+^T cells, and those that are recent migrants from the thymus express CD31 (123, 396).

## Conclusions- The Full Nature of Alloantigen Specific Treg Remains to Be Fully Resolved

For over 60 years the concept of “clonal deletion” has dominated the mechanism of self- non self and transplant tolerance. At the time the theory was proposed, there was no knowledge of T cells or regulatory processes. The prime role of peripheral T cells, not antibody, in allograft rejection was not appreciated.

The study of T cells, led to the discovery of numerous pathways for the activation of effector T cells to distinct functional subtypes including Th1, Th2, Th17, Tfh. Suppressor T cells were described early in the T cell era but the reliance on CD8 and I-J as markers of these cells led to a belief that suppression was an artefact. Suppressor T cells were taboo from the mid 1980s until the early 2000s.

Our work on alloantigen specific T regulatory cells in transplant tolerance identified they were CD4^+^CD25^+^T cells and were alloantigen-specific. FoxP3 expression is essential for functioning Treg (397) and the induction of transplant tolerance (398). Studies of activated alloantigen-specific Treg were difficult as they die rapidly *ex vivo* even if stimulated by specific alloantigen (150, 340, 345). Recently others have reported that activated CD4^+^CD25^hi^FoxP3^hi^CD45RA^-^Treg die and are hard to get to proliferate (349, 399). What promotes the survival and function of these activated Treg, is a key question to be resolved to maximize their use in promoting transplant tolerance.

Our studies described above are one of the few to have addressed this question, and identify at least three cytokines (IL-12, IFN−γ and IL-5) produced late in the immune response by effector cells. These cytokines appear after production of early cytokines such as IL-2 and IL-4 wanes. It is in this late chronic phase of the allograft response that the activated effector cells produce cytokines that activate alloantigen specific Treg that mediate transplant tolerance by inhibition of the rejection response at the site of inflammation.

At present and for the last 25 years, studies on Treg have focussed on resting naïve Treg. These cells can be expanded by the presence of IL-2 or IL-4, and possibly other cytokines that are yet to be defined. The Treg that mediate transplant tolerance die without activation by specific alloantigen and cytokines produced by the ongoing effector response to the allograft. They do not mature in the presence of IL-2, and do not need IL-2 to survive.

While cytokines such as IL-2 and IL-4 activate naïve CD4^+^CD25^+^Foxp3^+^Treg they cannot sustain the highly potent Treg, which become dependent on cytokines produced in the late stages of activation of effector T cells when production of IL-2 and IL-4 wanes. In Type 1 responses, these are IFN-γ and IL-12p70. In Type 2 responses, IL-5 continues to be produced as does IL-13, both of which are anti-inflammatory (358, 361).

The early studies on neonatal thymectomy unmasked a dual and parallel function of the thymus, first producing effector T cells that were not fully deleted of auto-reactive clones, and a few days later releasing T cells that suppress autoimmunity. We now know these inhibitors of autoimmunity are naïve CD4^+^CD25^+^FoxP3^+^Treg. In neonatal tolerance induction, the alloantigen could selectively activate the newly produced Treg to suppress the allograft response. There were early cues that neonatal tolerance was in part maintained by inhibitory forces. It could be argued that tolerance induction in neonates uses the same processes that protect against autoimmunity, where CD4^+^CD25^+^FoxP3^+^Treg control the activation of auto reactive cells that are not deleted during ontogeny.

There are many other immune mechanisms that can come into play, including response of the graft, loss of donor antigen presenting cells, and overactivation of the immune response, leading to exhaustion. We are still some way from understanding all these mechanisms, especially the multiple pathways of activation of naïve Treg, We recently reported that naïve CD4^+^CD25^+^Treg cultured with IL-2 and alloantigen are induced to express CD8 as well as CD4, and the CD4^+^CD8^+^T cells are the potent alloantigen specific Treg (370). This finding raises the possibility naïve CD4^+^CD8^-^CD25^+^FoxP3^+^Treg could produce CD8^+^Treg. Many other types of regulatory cells have been described but our focus was on alloantigen specific CD4^+^CD25^+^FoxP3^+^Treg as this is the most common and dominant regulatory cell.

It is increasingly apparent that specific alloantigen activated CD4^+^CD25^+^FoxP3^+^Treg, not naïve Treg mediate alloantigen specific transplant tolerance. How to induce them and monitor them remains a challenge. Harnessing the potent antigen-specific Treg, may lead to tolerance to grafts in patients. It is also apparent that in many models of Transplant Tolerance, clonal deletion is not present and is not necessary.

Within the heterogenous populations of CD4^+^CD25^+^FoxP3^+^CD127^lo^Treg, the highly activated Treg express more CD25 and FoxP3. These cells die and are thought not to proliferate, leading to the belief they serve little or no function. This has parallels with Medawar and Florey’s dismissal of small lymphocytes and thymus derived cells, mentioned above. More intense study of these cells may draw us closer to solving how to induce transplant tolerance.

## Author Contributions

BH, NV, GT, and SH all contributed to the design, writing, proof reading of the manuscript. All authors contributed to the article and approved the submitted version.

## Funding

The work was supported by Liverpool Hospital and University of New South Wales Sydney.

## Conflict of Interest

The authors declare that the research was conducted in the absence of any commercial or financial relationships that could be construed as a potential conflict of interest.

## Publisher’s Note

All claims expressed in this article are solely those of the authors and do not necessarily represent those of their affiliated organizations, or those of the publisher, the editors and the reviewers. Any product that may be evaluated in this article, or claim that may be made by its manufacturer, is not guaranteed or endorsed by the publisher.

## Abbreviations

ALS, Anti-lymphocyte serum; CML, Cell mediated lysis; CSA, Cyclosporine A; CTL, Cytotoxic T cell; GVH, Graft versus host; mAb, Monoclonal antibody; MHC, Major histocompatibility complex; MLC, Mixed lymphocyte culture; TDL, Thoracic duct lymphocytes; Treg, T regulatory cell.
