# Supplementary material for: Transplant Tolerance, Not Only Clonal Deletion
Source: Front Immunol. 2022 Apr 21;13:810798. doi: 10.3389/fimmu.2022.810798 (PMC9069565; doi:10.3389/fimmu.2022.810798)
Supplement: Supplementary file 1 [file Table_1.pdf]

Supplementary Table 1. Observations that supported clonal deletion in utero and in neonates

1936 Taub (2, 5). Viral infection in utero, leads to no immune elimination after birth.

1945 Owen (3, 4). Freemartin Cattle share a placenta and have haemopoietic chimerism through life.

1943 Gibson and Medawar (14). In a burn patient, skin grafts from a brother do not survive and second graft from the same donor was destroyed faster.

1947 Fagraeus. Described plasma cell expansion in spleen after repeated intravenous injection of homologous antigen (60). Plasma cells produce antibody.

1949 Burnet and Fenner. *During foetal life, there is an inability to react to antigen and produce antibodies by the action of the embryo's own tissue on the reticuloendothelial system (2).*

1950 Burnet, Stone and Edney (2): *" Injection of foreign red cells, bacterial virus or living influenza virus into chick embryos did not provoke antibody production. No change in the subsequent response of the hatched chick to the corresponding antigen could be demonstrated."* No clonal deletion or tolerance was demonstrated.

1951 Anderson, Billingham, Lampkin, Medawar (18). Dizygotic cattle twins accept skin grafts from each other.

1953 Hasek. After parabiosis of chickens in egg the haemagglutinin response to the opposite chicken were suppressed (37) and skin from the parabiosis partner was accepted (37, 38).

1953 Billingham, Brent, Medawar (25). Neonates injected with allogeneic cells, later in life accepted allografts from the same strain.

1958 Nossal and Lederberg (9, 10). Each cell/clone produced only one antibody.

1960 Medawar and Burnet, share Nobel Prize *"for discovery of acquired immunological tolerance".*

1961 Miller (86). Neonatal thymectomy prevents rejection and leads to a transplant tolerant state- first identification of T cells.

1961 Gowans (13, 49). Lymphocytes mediate immune responses.
