# Supplementary material for: Transplant Tolerance, Not Only Clonal Deletion
Source: Front Immunol. 2022 Apr 21;13:810798. doi: 10.3389/fimmu.2022.810798 (PMC9069565; doi:10.3389/fimmu.2022.810798)
Supplement: Supplementary file 2 [file Table_2.pdf]

Supplementary Table 2. Discoveries related to suppressor regulatory T cells

1970 Suppressor T cells (139).

1975 Neonatal transplant tolerance transferrable by chimeric T cells (187).

1975 Adult transplant tolerance transferred by lymphocytes (276).

1984 Normal thymocytes suppress rejection (332).

1984-5 Tolerance transferred by antigen specific $CD4^+$T suppressor cells (149).

1986 Th1 and Th2 cells, interact (351).

1988 Tolerant $CD4^+$T cells are short-lived cells (340).

1990 Tolerance mediated by $CD4^+CD25^+CD45RC^+$ Class II MHC^+T cells (150).

1993 Transferred tolerant $CD4^+$T cells induce host cells to become regulatory (282) "Infectious Tolerance".

1993 Tolerant $CD4^+$T cells cultured with alloantigen only survive with cytokines, including IL-2 (345).

1995 Thymic derived $CD4^+CD25^+$T cells suppress autoimmunity and rejection(151).

2002 Tolerance mediating Treg are in tolerant grafts (339)

2003 FoxP3 controls regulatory $CD4^+CD25^+$T cell development (397).

2005 Ex vivo expanded $CD4^+CD25^+$Treg can promote transplant tolerance (1).

2009 naïve Treg activated by alloantigen and IL-2 are more potent than naïve Treg at suppressing rejection (364, 367).

2009 Th1 and Th2 responses induce separate pathways of Treg activation (364).

2009 Treg population is heterogenous comprising of resting ($CD45RA^+$), activated ($CD45RA^-CD25^{hi}Foxp3^{hi}$) and cytokine secreting Treg (349).

2014 IL-12p70 induces IL-2 and alloantigen activated Treg to potent Th1-like Treg (367).

2014 Complex nature of activation and survival pathways for Treg mediated by different Th1, Th2, Th17 and Tfh responses. IL-15 (379), TGF- β (380), IFN-γ (381), IL-12 (360), IL-4 (364) , IL-5 (369), IL-27 (382), IL-33 (383), IL-35 (384).

2017 Tolerance mediating alloantigen specific Treg maintained by IL-5(363) or IFN-γ (362).

2017 $CD4^+CD25^+$T cells from tolerant hosts do not proliferate to specific donor in the absence of IFN-γ, IL-5 and IL-12 (312).
